# Rotavirus NSP2: A Master Orchestrator of Early Viral Particle Assembly

**DOI:** 10.3390/v16060814

**Published:** 2024-05-21

**Authors:** Sarah L. Nichols, Cyril Haller, Alexander Borodavka, Sarah M. Esstman

**Affiliations:** 1 Department of Biology, Wake Forest University, Wake Downtown, 455 Vine Street, Winston-Salem, NC 27106, USA; nichsl20@wfu.edu; 2 Department of Chemical Engineering and Biotechnology, Cambridge University, Philippa Fawcett Drive, Cambridge CB3 0AS, UK; cjh253@cam.ac.uk

**Keywords:** rotavirus, nonstructural protein 2, viroplasm, particle assembly, genome segment assortment, genome packaging, replication

## Abstract

Rotaviruses (RVs) are 11-segmented, double-stranded (ds) RNA viruses and important causes of acute gastroenteritis in humans and other animal species. Early RV particle assembly is a multi-step process that includes the assortment, packaging and replication of the 11 genome segments in close connection with capsid morphogenesis. This process occurs inside virally induced, cytosolic, membrane-less organelles called viroplasms. While many viral and cellular proteins play roles during early RV assembly, the octameric nonstructural protein 2 (NSP2) has emerged as a master orchestrator of this key stage of the viral replication cycle. NSP2 is critical for viroplasm biogenesis as well as for the selective RNA–RNA interactions that underpin the assortment of 11 viral genome segments. Moreover, NSP2’s associated enzymatic activities might serve to maintain nucleotide pools for use during viral genome replication, a process that is concurrent with early particle assembly. The goal of this review article is to summarize the available data about the structures, functions and interactions of RV NSP2 while also drawing attention to important unanswered questions in the field.

## 1. Introduction

Rotaviruses (RVs) are 11-segmented, double-stranded (ds) RNA viruses belonging to the *Sedoreoviridae* family within the *Reovirales* order [1]. Nine genetically divergent species of RV (groups A-D and F-J) have been discovered to date; however, human infections are typically caused by group A strains [2,3]. Notably, group A RVs induce severe, dehydrating diarrhea and vomiting in infants and young children, which can be life-threatening in the absence of medical care [4]. Despite the availability of several live-attenuated vaccines against human group A RVs, it is estimated that infections still lead to ~128,000 child deaths each year in developing world regions [4]. Continued research on RVs is warranted because the outcomes of such work may inform new treatment measures to prevent childhood diarrhea. In addition to infecting humans, group A RVs also infect a broad range of avian and mammalian hosts in nature [5]. Because human strains are poorly cultivatable, the field has predominantly relied on animal strains as models (e.g., simian strains SA11 and RRV, bovine strains UK and RF, and porcine strain OSU) [6]. Structural and functional studies of these model strains and their individual viral proteins have shed light on many aspects of RV biology. In particular, the RV nonstructural protein 2 (NSP2) has been investigated by a cadre of laboratories over the past several decades. Results of this work have revealed multi-faceted roles for this protein during viral replication, particularly during the early stages of particle assembly. This review article seeks to summarize the growing body of published literature on RV NSP2 as well as emphasize the key gaps in knowledge for future research studies.

## 2. Virion, Genome and Replication Cycle

**Figure 1:**
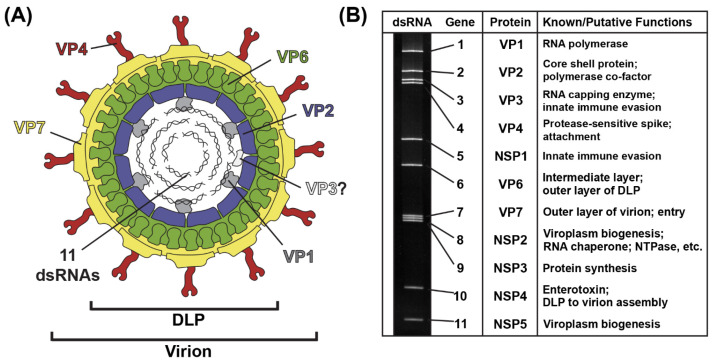
**Rotavirus Virion, Genome and Proteins.** (**A**) Cartoon image of the infectious rotavirus virion, which is made up of six viral proteins (VP1–VP4, VP6 and VP7). The location of VP3 is unknown. The 11 dsRNA genome segments are encased within the particle. (**B**) Rotavirus strain SA11 dsRNA genome segments are shown separated in a polyacrylamide gel. Each gene is numbered, and the encoded protein and putative/known functions are listed to the right.

The RV virion is a ~100 nm non-enveloped, triple-layered particle that exhibits icosahedral symmetry (Figure 1A) [7,8]. The outermost layer of the virion is comprised of the VP7 glycoprotein, and it is embedded with numerous copies of the VP4 spike protein [7,8]. Proteolysis of VP4 by trypsin-like enzymes is required for RV attachment to cells and results in the formation of VP8* and VP5* fragments [9]. The intermediate layer of the virion is made up of VP6, and the innermost core shell is comprised of VP2 [7,8]. Within the VP2 core shell reside several copies of the VP1 RNA-dependent RNA polymerase and the VP3 RNA capping enzyme [8,10,11]. While the position of VP3 inside of the particle has not yet been validated, VP1 is bound beneath the VP2 core shell, just off-center from each icosahedral fivefold axis [8,11]. Also located within the VP2 core shell are the 11 dsRNA genome segments, which range in size from ~0.5 kb to ~3.3 kb, together comprising a ~18–23 kb viral genome [12] (Figure 1B). The extreme 5′ and 3′ termini of the 11 dsRNA genome segments are conserved, and they abut more variable non-coding regions (NCRs) [12]. For each segment, the NCRs surround a central open-reading frame (ORF) that typically encodes a single viral protein [12]. As such, most group A RV strains express 11 proteins: (i) 6 structural proteins (VP1–VP4, VP6 and VP7) that make up the virion particle and (ii) 5 nonstructural proteins (NSP1-NSP5) that play various roles during the viral replication cycle but are not incorporated into particles [12] (Figure 1B). Some group A RV strains express an additional protein (NSP6) from an alternative ORF in the NSP5-coding gene [12].

**Figure 2:**
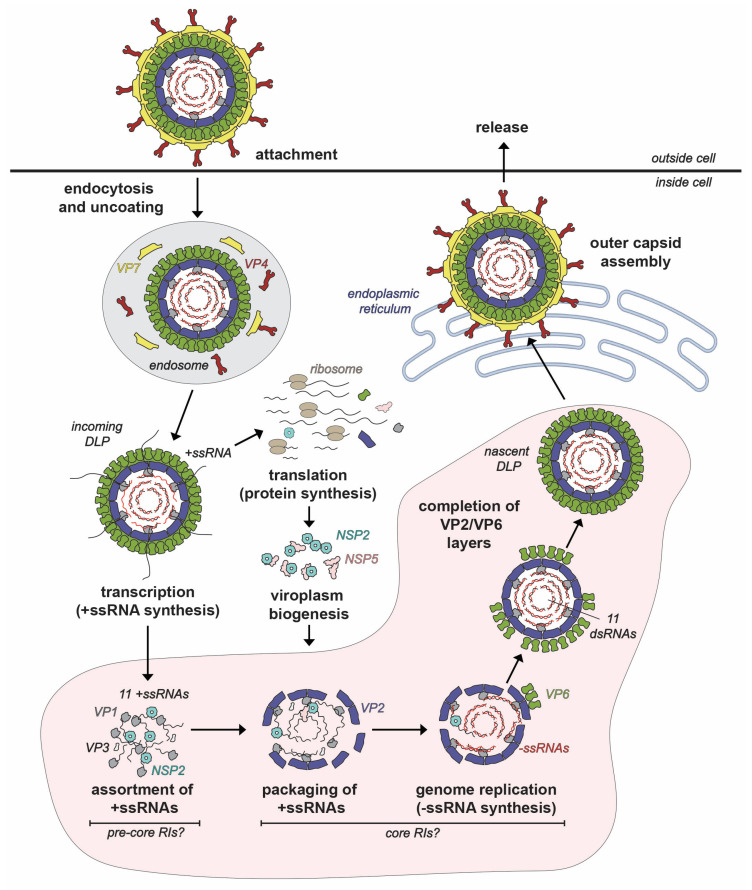
**Rotavirus Replication Cycle.** The rotavirus virion attaches to the host cell and enters via endocytosis. The outer VP7-VP4 layer is removed during the entry process, resulting in a transcriptionally active double-layered particle (DLP). Transcripts (+ssRNAs) first serve as mRNAs for protein synthesis. Once made, nonstructural proteins NSP2 and NSP5 interact to form viroplasms, where the early stages of particle assembly occur. The hypothetical steps and putative assembly intermediates involved in early assembly inside viroplasms are labeled. Specifically, the +ssRNAs recruited to the viroplasm are thought to be assorted in the context of pre-core RIs, and then they are packaged into VP2-containing assembly intermediates called core RIs. In this core RI context, the +ssRNAs are used for −ssRNA (red) synthesis by VP1 to recreate the dsRNA genome segments inside a particle that morphs into a DLP. Nascent DLPs made in viroplasms acquire their outer VP7-VP4 layer in the endoplasmic reticulum prior to exiting from the cell.

Like all members of the *Reovirales* order, the RV replication cycle is entirely cytoplasmic [12] (Figure 2). In humans and lab animals, RVs primarily infect the epithelial cells of small intestinal villi [13,14]. However, most studies of RV replication are performed using transformed monkey kidney epithelial cell lines (e.g., MA104 and COS-7) [6,14]. RV infection is initiated by the binding of proteolytically activated virions (i.e., with cleaved VP4 spikes) to protein and/or carbohydrate receptors on the host cell surface [15]. The virus typically enters cells via receptor-mediated endocytosis [15,16,17]. In the low Ca^2+^ environment of the endosome, virions undergo shedding of their outer VP4/VP7 layer concurrent with penetration of the endosomal membrane, resulting in the deposition of double-layered particles (DLPs) into the cytosol [18,19,20]. As quickly as 15 min after penetration, the VP1 polymerases within the interior of the DLPs initiate viral RNA transcription [21]. More specifically, transcription is the synthesis of 11 different positive-sense, single-stranded RNA (+ssRNA) using the minus-strands (−ssRNAs) of the 11 dsRNA genome segments as templates [21]. The nascent +ssRNAs receive a 5′ 7-methylguanosine cap via the activities of VP3 prior to their egress from channels located at the fivefold axes of the capsid [21,22,23,24]. Serving as messenger RNAs (mRNAs), the +ssRNAs are translated into the 11 RV proteins by the host cell ribosomal machinery [25,26,27]. The exact sites of RV protein synthesis are unknown but likely include cytosolic polysomes and the rough endoplasmic reticulum (ER) [27].

Following protein synthesis, nonstructural proteins NSP2 and NSP5 nucleate the formation of cytoplasmic inclusions, called viroplasms, which are the sites of early RV particle assembly [28,29,30]. More simply, viroplasms can be thought of as “factories” wherein VP1, VP2, VP3, VP6 and the 11 viral RNA segments come together in a highly coordinated manner to form new DLPs (Figure 2). While the mechanistic details are incompletely understood, the DLP assembly process can be described as having 4 interconnected, synergistic steps: (i) assortment of the 11 distinct +ssRNAs, (ii) packaging of the 11 +ssRNAs along with the VP1/VP3 into a morphing VP2-containing particle, (iii) VP1-mediated −ssRNA synthesis (i.e., genome replication), converting the 11 +ssRNAs into the dsRNA genome segments and (iv) completed assembly of VP2 and VP6 capsid layers to create the intact DLP [18] (Figure 2). As we will detail in this review article, NSP2 plays several critical roles during early RV particle assembly—it nucleates viroplasms, mediates the assortment/packaging of the 11 +ssRNAs into the assembling capsid and may even help to maintain nucleotide substrate pools for genome replication. The final stages of RV particle assembly occur in the ER, where the VP4/VP7 capsid layer is added to newly formed DLPs, and the resulting virions exit from the cell via non-lytic or lytic pathways, depending on cell type [31,32].

## 3. Discovery and Early Characterization of NSP2

RV NSP2 was first described in the early 1980s when polypeptides from SA11-infected MA104 cells were analyzed via immunoprecipitation and partial proteolytic peptide mapping [33]. These experiments revealed the presence of a 35 kDa protein (originally called NS35 and later renamed NSP2) that failed to immunoprecipitate using antisera raised against DLPs, suggesting it to be a nonstructural protein [33]. Sequence analysis was used to deduce that the NSP2 protein was a conserved, highly basic protein and was coded for by strain SA11 segment 8; this assignment was experimentally confirmed by in vitro translation of viral mRNAs and RNA–RNA hybridization assays [34,35]. Following its discovery, multiple studies sought to better understand the possible function(s) of NSP2 during RV replication by investigating its intracellular localization and interactions. Immunocytochemistry and colloidal gold labeling of SA11-infected MA104 cells showed that NSP2 localized to viroplasms, which were judged by electron microscopy to be the sites of DLP assembly [36]. Moreover, NSP2 was reported to be a component of RV replication-assembly intermediates (RIs) that were purified from SA11-infected MA104 cells using either native gel agarose electrophoresis or immunoprecipitation [37,38,39,40]. Specifically, Patton and Gallegos reported that NSP2 was found in complexes called pre-core RIs, which lacked the VP2 core shell protein but contained VP1, VP3 and NSP5 [37]. NSP2 was also found in core RIs that contained VP2 along with the components of the pre-core RI [38]. Upon incubation with Mg^2+^ and NTPs, isolated core RIs (but not pre-core RIs) were found to be active for −ssRNA synthesis, creating the 11 dsRNA genome segments in vitro [38,39,40]. These results suggested that the assortment of the 11 genome segments (i.e., the 11 +ssRNAs), likely in the context of the pre-core RI, preceded core RI-mediated genome replication (Figure 2) [41]. Furthermore, these results indicated that the VP2 core shell protein was required for −ssRNA synthesis by the VP1 polymerase, a finding that has been since validated [42,43,44,45,46]. The observation that NSP2 was found in both pre-core RIs and core RIs suggested that it may function during the earliest DLP assembly steps, namely +ssRNA assortment/packaging and possibly genome replication. Supporting this notion were studies of a temperature-sensitive SA11 mutant virus that has a lesion mapping to segment 8 (*ts*E) [47]. *ts*E replicates well when grown in cell culture at the permissive temperature of 31 °C, but its replication is severely diminished at the non-permissive temperature of 39 °C [48,49]. Ramig and Petrie showed an increase in the formation of empty RV particles and a decrease in the number of viroplasms in *ts*E-infected MA104 cells at 39 °C versus 31 °C [49]. Moreover, *tsE* was reported to be phenotypically negative for −ssRNA synthesis at 39 °C, though it remains unclear whether this phenotype was an indirect consequence of failed +ssRNA packaging, which is pre-requisite to −ssRNA synthesis [50].

Early mechanistic insights into NSP2 functions were revealed by a series of biochemical studies. Kattoura et al. employed UV-crosslinking to demonstrate that the protein (i) assembles into higher-ordered oligomers in the cell, (ii) binds to +ssRNA (and to a lesser extent dsRNA) in a sequence-independent manner and (iii) interacts directly with the VP1 polymerase [51,52]. Both the oligomeric nature and RNA binding properties of NSP2 were confirmed by Taraporewala et al. using recombinant protein [53]. Specifically, by employing gel shift assays the authors were also able to show that recombinant, multimeric NSP2 bound to +ssRNA in discrete, cooperative steps [53]. Interestingly, the same authors also showed that the octameric form of NSP2 exhibited a Mg^2+^-dependent nucleotide triphosphatase (NTPase) activity, meaning that it could hydrolyze the γ-phosphate from NTP to create an NDP in vitro [53,54]. Based on this result, the NTPase activity of NSP2 was hypothesized to serve as a source of energy for incorporating the 11 +ssRNAs into morphing RV particles [53,54]. Schuck et al. built upon the work of Taraporewala et al. and used a variety of biophysical techniques to confirm the octameric status of NSP2, and to show that it undergoes conformational changes upon binding to RNA, Mg^2+^ and NTP/NDPs [55]. In addition, NSP2 was reported to have RNA helix-destabilizing activity, as it disrupted RNA–RNA duplexes in vitro [56]. This function was predicted to be important for unwinding stem-loop structures in viral +ssRNAs during assortment and packaging [56]. Finally, NSP2 was reported to bind directly to NSP5, and co-expression of these two proteins alone induced the formation of viroplasm-like structures in the absence of infection [57,58]. Thus, in addition to roles during the DLP assembly pathway itself, NSP2 also seemed to help build the factories within which such assembly occurred. Altogether, these early studies created a strong foundation for the following decades of structural and functional work, revealing deeper insights into NSP2’s multifaceted roles during RV replication.

## 4. Structural and Enzymatic Studies of NSP2

**Figure 3:**
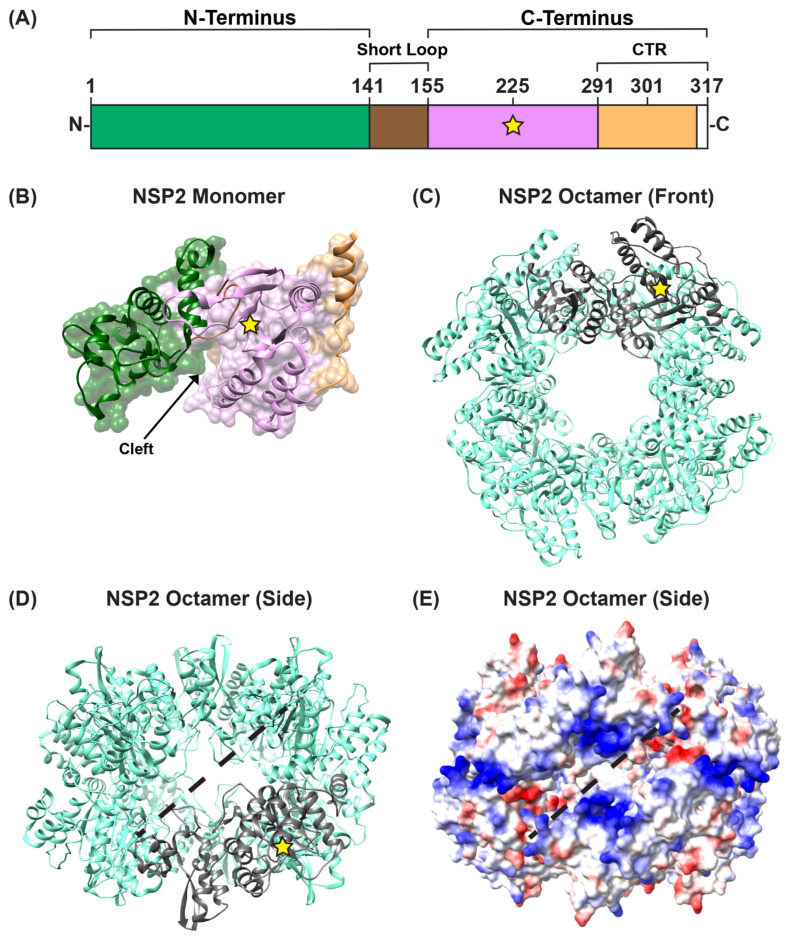
**NSP2 Structure.** (**A**) Linear schematic of strain SA11 NSP2 (317 amino acids in length). The protein is comprised of two domains: an N-terminal (green) and a C-terminal domain (pink), separated by a short loop (brown). The extreme C-terminal region (CTR; residues 291–317) is represented in orange; however, CTR residues 314 to 317 are unstructured (white). A yellow star represents the catalytic site H225. (**B**) SA11 NSP2 monomer (PDB no. 1L9V) is colored as in panel (**A**) and is shown in both surface and ribbon representation. An arrow indicates the electropositive cleft, and a yellow star represents the catalytic site H225. (**C**) SA11 NSP2 octamer structure (PDB no. 1L9V) is shown in ribbon representation (cyan), which a single monomer highlighted in gray. (**D**) SA11 NSP2 octamer from panel (**C**) is flipped “forward” 90 degrees to show the side view along the two-fold axis. The electropositive groove that comprises RNA/NSP5 binding site is shown as a dashed line. (**E**) SA11 NSP2 octameric structure from panel (**D**) is shown in electrostatic surface representation. Red indicates negative charge, while blue indicates positive charge.

Several structures of recombinant, octameric group A NSP2 have been solved by either X-ray crystallography or by using single particle cryo-EM reconstruction techniques [59,60,61,62,63,64]. These studies show that each NSP2 monomer within the octameric unit has an N-terminal domain (residues ~1–140) and a C-terminal domain (residues ~156–313) that are connected by a short loop (residues ~141–155) (Figure 3A,B). Residues ~313–317 are unstructured (Figure 3A). The N-terminal domain is described as having two sub-domains: (i) the first is composed of two pairs of β-strands separated by two α-helices, and (ii) the second consists of four α-helices (Figure 3B). The two N-terminal subdomains are connected by a loop that is largely basic (Figure 3B). This loop and an α-helix from the N-terminal domain form a large portion of one side of a 25 Å-deep electropositive cleft on the monomer (Figure 3B). The C-terminal domain has a prominent twisted anti-parallel β-sheet followed by α-helices (Figure 3B). The other side of the electropositive cleft consists of a C-terminal α-helix and a loop that exists between residues ~248–265 (Figure 3B). The base of the cleft is composed of C-terminal anti-parallel β-strands (residues ~186–191; 226–230), along with a loop structure formed of residues ~221–226 (Figure 3B). The extreme C-terminal region (CTR; residues ~291–313) of NSP2 consists of a flexible linker region and a terminal α-helix (Figure 3A,B). The functional NSP2 octamer is formed via tail-to-tail stacking of two tetramers in a manner that creates a 35-Å wide central hole (Figure 3C,D).

**Figure 4:**
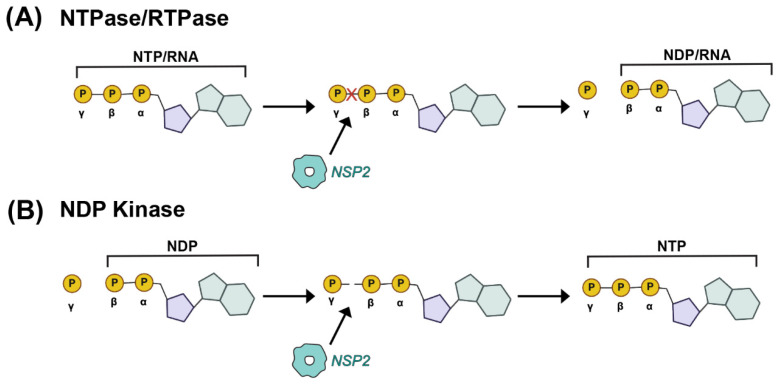
**Enzymatic Activities of NSP2.** (**A**) Nucleoside triphosphatase (NTPase) and RNA-triphosphatase (RTPase) activities of NSP2 are cartoon. In this case, the terminal γ phosphate of either a cellular NTP or a viral RNA is removed by NSP2, yielding inorganic phosphate and NDP. (**B**) Nucleoside diphosphate kinase (NDPase) activity of NSP2 is cartooned, whereby the terminal γ phosphate is added to a cellular NDP to form an NTP.

In the functional octamer, the electropositive clefts of NSP2 monomers come together in the context of two highly basic, 25 Å deep and 30 Å wide grooves that run diagonally across each tetramer–tetramer interface [59] (Figure 3D,E). The histidine triad (HIT)-like motif of NSP2, which includes the catalytic residue H225 that mediates the hydrolysis of the γ-phosphate from an NTP to create NDP, is accessed within this region of the protein [59,65] (Figure 3D and Figure 4A). Using structural and biochemical approaches, Kumar et al. showed that the hydrolyzed γ-phosphate is transferred to H225 of NSP2 [61]. The formation of this phosphohistidine intermediate was found to be part of an in vitro NDP kinase activity, whereby NSP2 converts NDP to NTP via transfer of the bound γ-phosphate [61] (Figure 4B). The observation that NSP2 has both NTPase and NDP kinase activities suggested a possible role for NSP2 in maintaining pools of nucleotides in the viroplasm for use during genome replication. It was further shown that recombinant NSP2 exhibits an in vitro RNA triphosphatase (RTPase) activity, whereby it hydrolyzes the γ-phosphate from an RNA molecule via a mechanism requiring H225 [62,65] (Figure 4A). Hu et al. investigated this RTPase mechanism by determining the X-ray crystal structure of recombinant NSP2 in complex with the 5′consensus sequence of RV −ssRNA (i.e., 5′GG) [62]. Consistent with previous studies, they found that the oligoribonucleotide interacted extensively with highly conserved residues in the enzymatic cleft of the monomers, which are accessed in the context of the grooves [62]. Indeed, cryo-EM reconstructions of NSP2 in complex with RNA support the notion that the grooves are also major RNA-binding sites [60]. Moreover, cryo-EM analysis of NSP2 in complex with a short fragment of NSP5 (residues 66–188) indicated that NSP5 may interact with NSP2 via the grooves, competing with RNA [60] (Figure 3D,E). The grooves have also been reported to bind tubulin, although it remains unclear whether NSP2 is involved in microtubule depolymerization [66]. It is possible that the highly charged nature of the groove and its geometry may accommodate the binding of multiple charged ligands, including small RNAs and negatively charged proteins.

**Figure 5:**
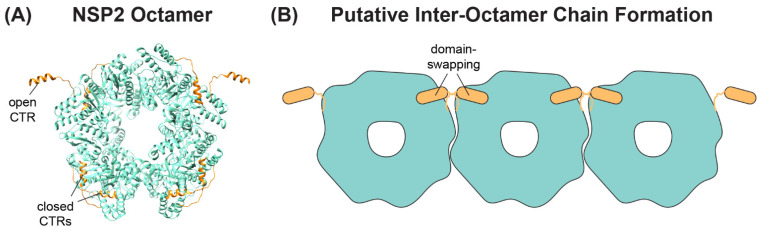
**NSP2 CTR Conformations and Putative Inter-Octamer Chains.** (**A**) SA11 NSP2 octamer (PDB no. 4G0A) from Hu et al. is shown in ribbon form (cyan), with the flexible CTRs highlighted in orange. Representative open and closed CTRs are labeled. (**B**) Cartoon image of putative NSP2 inter-octamer chains. Under some crystallography conditions, the open CTR of one octamer can interact with the body of a neighboring octamer (and vice-versa) via a domain-swapping interaction.

A separate, novel observation by Hu et al. was that the CTR of one NSP2 monomer in the octamer unit exhibited an “open” conformation that was flipped outward relative to the rest of the protein [62] (Figure 5A). The “open” conformation of the CTR was underpinned by flexible linker residues 293–295, allowing for a domain-swapping interaction that caused adjacent NSP2 octamers to interact within the crystal lattice [62] (Figure 5B). However, octamer chains were not found in the cryo-EM structures of NSP2, and it remains unknown whether such interactions occur in solution or in infected cells [64]. Still, as we discuss in detail in the following sections, the flexible CTR of NSP2 has emerged as an important functional domain of the protein for both viroplasm formation and for +ssRNA segment assortment.

## 5. Role of NSP2 in Viroplasm Formation

**Figure 6:**
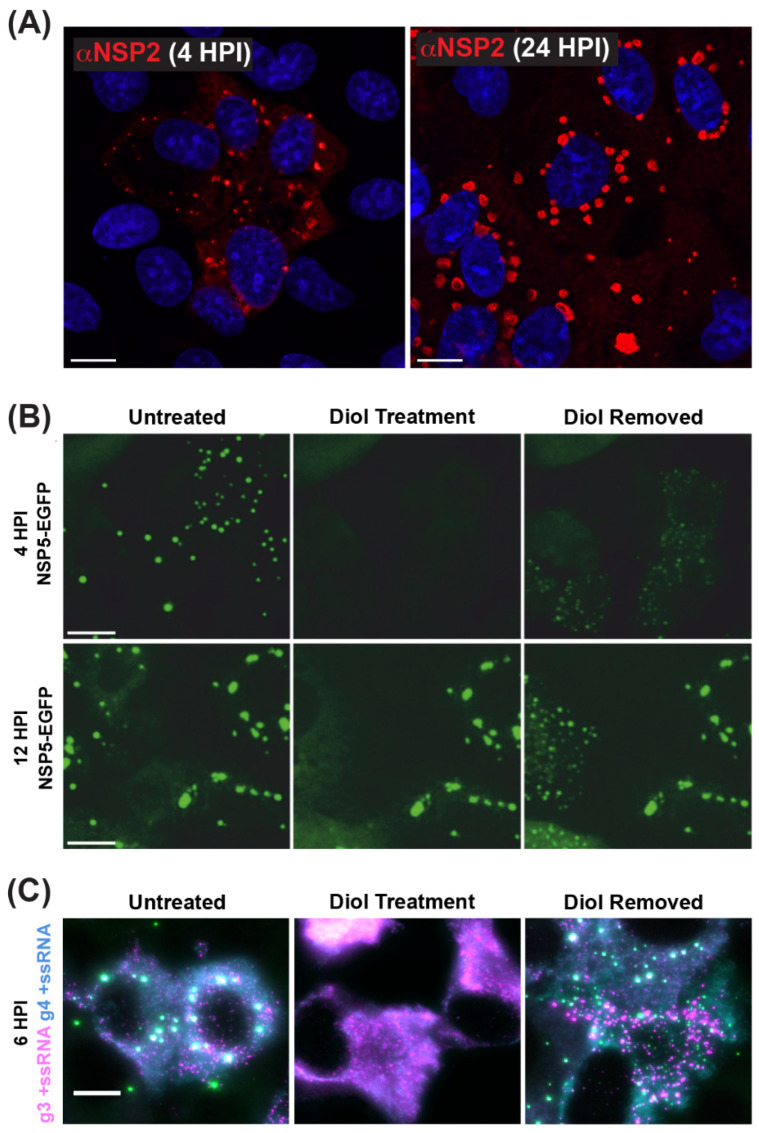
**Viroplasms are Cytoplasmic Biomolecular Condensates Formed via LLPS.** (**A**) Fixed immunofluorescence images of SA11-infected MA104 cells at 4 and 24 h post-infection (HPI). Viroplasms were stained with a polyclonal antibody against NSP2 (αNSP2; red), and cell nuclei were stained with Hoechst (blue). Scale bar = 10 μm. Images adapted from reference [70] with permission. (**B**) Viroplasms formed in MA104 cells stably expressing NSP5-EGFP and infected with strain SA11. Numerous small viroplasms can be dissolved when low concentrations of aliphatic diols (4.7% propylene glycol or 4% 1.6-hexane diol) are applied directly to the cell culture medium at 4 h post-infection (HPI). Removal of diols from the medium results in reassembly of multiple smaller granules dispersed in the cytosol (right panel). At 12 HPI, viroplasms become larger and less regular in shape. These larger viroplasms become resistant to the application of aliphatic diols. Scale bar = 50 µm. Images adapted from [71] with permission. Copyright Creative Commons Attribution License (https://creativecommons.org/licenses/by/4.0/). (**C**) RNA FISH imaging of gene segment 3 +ssRNA (magenta, g3 +ssRNA) and gene segment 4 +ssRNAs (cyan, g4 +ssRNA) in SA11-infected NSP5-EGFP-expressing MA104 cells fixed at 6 HPI. Viroplasms were treated with 4.7% (*v*/*v*) propylene glycol (middle) at 4 HPI, releasing +ssRNAs into the cytoplasm. These granules reformed after replacing the propylene glycol-containing cell culture medium, resulting in the rapid re-localization of g3 +ssRNA (magenta) and g4 +ssRNA (cyan) transcripts are detected via smFISH, and colocalizing RNAs (white). Scale bar = 10 µm. Images adapted from [72] with permission. Copyright Creative Commons Attribution License (https://creativecommons.org/licenses/by/4.0/).

As mentioned previously, viroplasms are cytosolic inclusions that serve as sites for the early stages of RV particle assembly, including the steps of +ssRNA assortment/packaging and −ssRNA synthesis (i.e., genome replication). Viroplasms can be seen microscopically as early as 2–4 h p.i., and they appear as small cytoplasmic puncta (~0.1–1 µm in diameter) [67,68,69,70] (Figure 6A). Over the course of infection, small viroplasms fuse together to become larger, with some reaching >5 µm in diameter [67,68,69,70]) (Figure 6A). Notably, Geiger et al. have shown that, at early stages, viroplasms are more dynamic and can be readily and reversibly dissolved by the low-concentration aliphatic diol treatments (e.g., 1,6-hexanediol or propylene glycol), suggesting that these structures are held by relatively weak, multivalent interactions between NSP2 and NSP5 (Figure 6B) [71]. Additionally, viroplasms contain all eleven types of +ssRNAs required for the assembly of nascent DLPs, as shown by multiplexed single-molecule fluorescence in situ hybridization (smFISH) analyses (Figure 6C) [72]. While viroplasms have also been shown to contain several viral and cellular proteins and lipids, NSP2 and NSP5 are sufficient for their nucleation [28,29,30,58,73]. Silencing of either NSP2 or NSP5 expression in RV-infected cells using RNA interference (RNAi) was shown to prevent viroplasm formation, further demonstrating their importance [74,75]. Interestingly, alanine mutation of the NSP2 catalytic histidine (H225A) did not affect the capacity of the protein to form viroplasms or viroplasm-like structures in cells [76,77]. Thus, the NTPase/RTPase/NDP kinase activities of NSP2 appear to be dispensable for viroplasm assembly.

**Figure 7:**
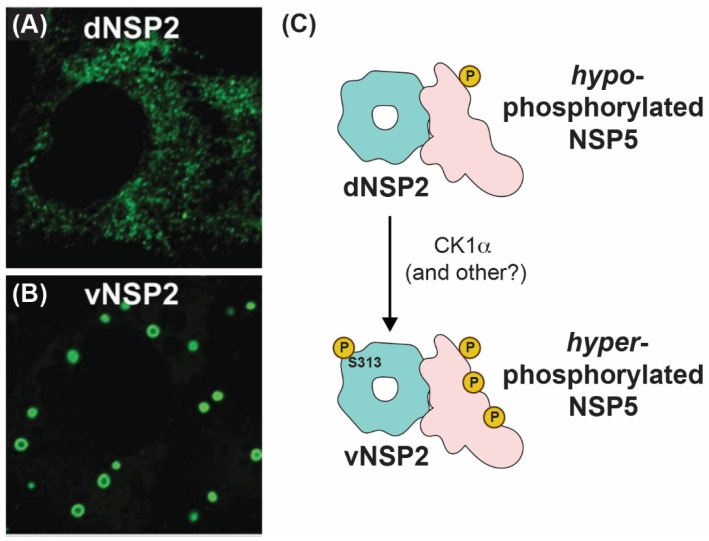
**Intracellular Localizations and Interactions of vNSP2 and dNSP2.** Fluorescence confocal micrographs of SA11-infected cells stained with monoclonal antibody against dNSP2 (**A**) and vNSP2 (**B**). Images taken from reference [78] with permission. (**C**) Cartoon model of vNSP2 and dNSP2 phosphorylation status and interactions with NSP5.

How NSP2 nucleates viroplasms alongside NSP5 has been an active area of research in recent years. Using conformation-specific monoclonal antibodies (mAbs), Criglar et al. showed that two forms of NSP2 are present in RV-infected cells [78]. Specifically, a diffuse form of NSP2 (dNSP2) predominantly localizes in the cytosol, while another form of NSP2 (vNSP2) accumulates in viroplasms [78] (Figure 7A,B). The authors show evidence to support the notion that dNSP2 interacts primarily with a hypo-phosphorylated NSP5, while vNSP2 interacts with a hyper-phosphorylated form of NSP5 [78] (Figure 7C). Phosphorylation of serine 313 (S313) in the flexible C-terminus of NSP2 itself was found to be enriched in the vNSP2 preparation, suggesting that it might contribute to differential mAb recognition [78] (Figure 7C). Further structural characterization of the used mAbs and their modes of recognizing distinct NSP2 conformations would substantiate the proposed model. A separate study performed by the same group provided experimental evidence that S313 phosphorylation of NSP2 was mediated by cellular casein kinase 1 (CK1α) [79]. Interestingly, silencing of CK1α resulted in vNSP2 displaying a diffuse phenotype similar to that of dNSP2 [79]. This result led to the hypothesis that S313 phosphorylation might underpin the switch from dNSP2 to vNSP2. It should be noted that silencing of CK1α is also known to inhibit NSP5 phosphorylation, also resulting in similar viroplasm dispersal and morphology alteration [80]. However, a mutant SA11 RV bearing a phosphomimetic change at NSP2 position 313 (S313D) still produced dNSP2, indicating that the conformation change between these two forms of the protein may be more complicated than this single post-translational modification [63]. Nevertheless, S313 appears to be important for proper viroplasm formation, as the S313D mutant produced phenotypically “disorganized” viroplasms early times p.i., and it was delayed in its overall replication [63]. Several studies have shown that lipid droplet markers co-localize with viroplasms, and Criglar et al. revealed that phosphomimetic S313D NSP2 physically interacts with lipid droplet proteins [63,81,82,83]. Thus, post-translational modifications of NSP2, such as phosphorylation (and possibly other modifications), appear to regulate NSP2 interactions with NSP5 and with cellular proteins during viroplasm biogenesis [28,63,84].

The NSP2 CTR is clearly an important determinant for viroplasm formation. Deletion of nearly the entire CTR (residues 293–301) abrogates the efficient formation of viroplasm-like structures in NSP2/NSP5 co-expressing cells [70,78]. As mentioned previously, the NSP2 CTR has the capacity to adopt an “open” conformation and participate in a domain-swapping interaction that links several octamers together, at least under crystallography conditions [62]. While not recapitulating the “open” NSP2 CTR, the crystal structure of S313D NSP2 showed enhanced lattice formation due to a hydrogen bond between D313 and R287 of a neighboring octamer [63]. Moreover, an SA11 RV bearing a lysine-to-glutamic acid change at the C-terminal position 294 (K294E) in NSP2 was found to exhibit viroplasm defects, including smaller and more numerous viroplasms under fixed- and live-cell conditions, as well as a delay in viroplasm fusion [70]. Residue K294 is located in a linker region that mediates the “open” vs. “closed” conformations of the CTR [62,70]. Molecular dynamics simulations of the K294E NSP2 monomer structure suggested that the mutation may have altered CTR flexibility, which in turn could have impacted inter-octamer associations [70]. Still, the proposed role of inter-octamer interactions observed in the crystal structure of NSP2 in the formation of viroplasms remains unclear and will require further investigation.

While the importance of NSP2 in the formation of viroplasms is undeniable, it is NSP5 that constitutes the primary component of viroplasms [85,86,87]. Using quantitative Western blotting of SA11 RV-infected cells, Geiger et al. estimated that the intracellular concentration of NSP5 exceeds that of NSP2, reaching >10 μM within 6 h p.i. [71]. NSP5 is a 22 kDa, serine/threonine-rich, intrinsically disordered and relatively acidic protein that can assemble into several higher-order oligomers [88,89,90]. During infection, this protein is O-glycosylated and differentially phosphorylated, causing it to migrate in sodium dodecyl sulfate (SDS)-polyacrylamide gels as phosphoisoforms ranging in size from 26 to 35 kDa [88,89,90]. Phosphorylation of NSP5 is required for proper viroplasm morphology and for viral replication [80,91,92]. Previous studies have shown that recombinant NSP5 can be phosphorylated by CK1α at serine 67 (S67), and only the hypo-phosphorylated isoform of NSP5 is observed in CK1α silenced cells [63,80]. However, further experiments revealed that CK1α is not sufficient for NSP5 hyper-phosphorylation [80]. Interestingly, only when NSP2 is co-expressed with NSP5 do the hyper-phosphorylated NSP5 isoforms appear, and in NSP2-silenced cells, only hypo-phosphorylated NSP5 is observed [57,63,93]. While it was proposed that NSP5 may undergo low levels of auto-phosphorylation through its autokinase activity, its phosphorylation was increased almost 5–10-fold upon incubation with recombinantly expressed NSP2 [76]. Interestingly, NSP2 mutants lacking NTPase activity were still capable of promoting NSP5 phosphorylation [76]. This result suggests that the mechanism of NSP5 phosphorylation may be linked to the conformational changes in NSP5 upon binding of NSP2, independent of its enzymatic activities. While an NSP2/NSP5 phosphorylation cascade is critical for viroplasm formation in infected cells, it is dispensable for viroplasm-like condensate formation in cells and in vitro [71,78]. Thus, phosphorylation may represent a regulatory mechanism controlling the timing of viroplasm formation, but it may not necessarily be a biophysical requirement for structure formation (see also below).

**Figure 8:**
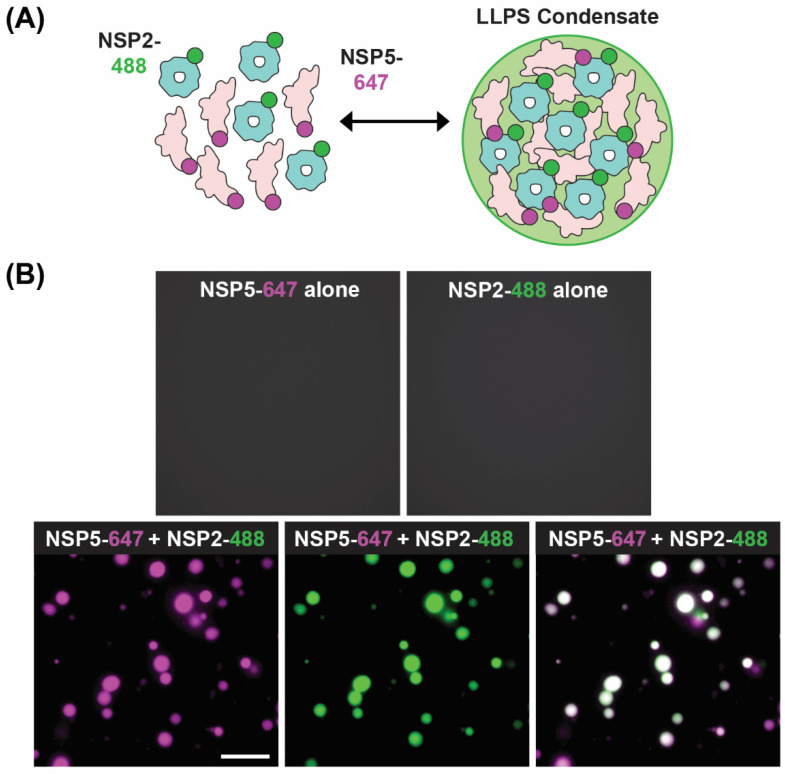
**In Vitro LLPS Biomolecular Condensate Assay.** (**A**) Recombinantly expressed, Atto 647-dye-labelled NSP5 (NSP5-647; magenta) and Atto488-dye-labelled NSP2 (NSP2-488; green) are mixed together in vitro to form droplets with LLPS characteristics. (**B**) Images showing either no condensate formation with individual proteins (top images) but efficient condensates when NSP5-647 and NSP2-488 are mixed (bottom images). Images modified from reference [71] with permission. Copyright Creative Commons Attribution License (https://creativecommons.org/licenses/by/4.0/).

A recent model of viroplasm formation proposes that these replicative factories are formed via the process of liquid–liquid phase separation (LLPS), primarily driven by interactions between NSP2 and NSP5 [71]. Notably, Geiger et al. showed that recombinant NSP2 and NSP5 spontaneously form droplets in vitro with biophysical properties of LLPS condensates (Figure 8). Further evidence for RV viroplasms being biomolecular condensates formed via LLPS includes (i) the ability of these organelles to fuse and relax into larger droplets, (ii) the fast recovery after photobleaching of fluorescently labeled NSP5 and NSP2 and (iii) rapid and reversible dissolution of droplets upon treatments of RV-infected cells with low concentrations of aliphatic diols, including 1,6-hexanediol and 1,2-propanediol [71] (Figure 6B). Interestingly, the ability of aliphatic diols to dissolve viroplasms instantly and reversibly in cells decreased over the course of infection, with larger, less round viroplasms being resistant to these solvents [71]. The loss in sensitivity towards the compounds that interfere with weaker hydrophobic and Van der Waals interactions indicates changes in the nature of biomolecular interactions, potentially reflecting changes in the apparent affinities between NSP2 and NSP5 as viroplasms mature [71]. These findings suggest that viroplasms are RNA-rich biomolecular condensates that are nucleated by NSP5 (containing mostly intrinsically disordered regions) and the RNA-binding protein NSP2.

Consistent with their liquid-like behavior, viroplasms initially coalesce over the course of infection and increase in size, with some reports suggesting that they migrate toward the perinuclear space [69,70,71]. Through experiments with microtubule destabilizing drugs, the movement and, ultimately, the fusion and condensation of viroplasms to the perinuclear space were inhibited, suggesting a role of microtubules in the movement and fusion of these structures [69,94]. Several studies corroborate this idea through immunofluorescence and electron microscopy, whereby viroplasms were shown to co-localize with microtubules [66,69,94]. This notion was further supported by co-immunoprecipitation experiments showing that NSP2 interacted with tubulin and dynein intermediate chain [95]. Thus, it is likely that the movement and coalescence of viroplasms over the course of infection is mediated by NSP2 directly interacting with dynein and kinesin motors; however, the functional significance of these interactions for RV replication remains unclear.

## 6. Role of NSP2 during +ssRNA Assortment

**Figure 9:**
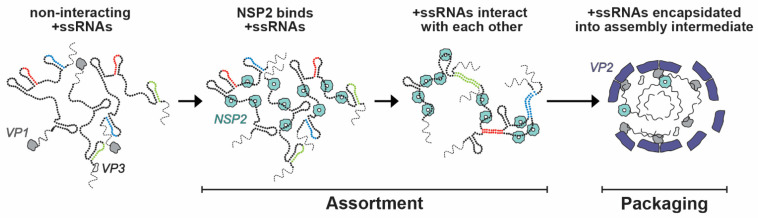
**Model of NSP2-dependent +ssRNA Assortment.** RV +ssRNAs (here only 3 RNAs are shown schematically) do not form stable RNA–RNA contacts with each other. NSP2 binding to +ssRNAs results in their structural rearrangements concomitant with the exposure of otherwise sequestered complementary sequences (interspersed sequences shown in red, blue and green) capable of inter-segment base-pairing. The exposed complementary sequences form stable sequence-specific inter-segment contacts (RNA helices shown in red, green and blue) during the +ssRNA assortment process. The resulting multi-RNA ribonucleoprotein complex (containing all 11 +ssRNAs) would then be encapsidated by a VP2 core assembly intermediate for genome packaging. Image modified from reference [96] with permission. Copyright Creative Commons Attribution License (https://creativecommons.org/licenses/by/4.0/).

Within the viroplasm itself, NSP2 likely plays several key roles, particularly at the step of +ssRNA (i.e., genome segment) assortment/packaging in pre-core and core RIs. In particular, the assortment of distinct +ssRNAs is thought to be mediated by the RNA chaperoning activity of NSP2, including RNA helix destabilizing and RNA annealing activities [56,64,96]. NSP2 can bind both folded RNA stem-loops and less structured RNAs, exhibiting a helix-destabilizing activity that is independent of cofactors and energy requirements (i.e., Mg^2+^ or ATP) [56,64]. Originally, NSP2 was proposed to remove secondary structures in +ssRNAs that would impede their packaging into core RIs as well as their replication into dsRNA [56]. However, recent investigations of NSP2 uncovered its RNA strand annealing activity in the context of long +ssRNAs in vitro, which may be conducive to the formation of an RNA assortment complex containing all 11 distinct +ssRNAs [96] (Figure 9). Specifically, Borodavka et al. showed that when incubated together, +ssRNAs did not interact unless NSP2 was added in molar excess over +ssRNAs [96]. To confirm that the +ssRNAs were interacting due to the inter-molecular base-pairing, the authors removed NSP2 after the addition to the +ssRNA samples via proteinase K digestion [96]. They found that the formation of inter-segment RNA–RNA complexes was indeed mediated by NSP2 that can be removed afterwards, consistent with its role as an RNA chaperone [96]. Importantly, the RNA chaperone activity of NSP2 was unaffected by the addition of ATP to reactions, which suggested that it was independent of NTPase activity [96]. Collectively, this data provides evidence that sequence-specific inter-molecular base-pairing, mediated by NSP2 binding to viral +ssRNAs, governs inter-segment RNA interactions and suggests that NSP2 acts as a viral RNA chaperone (Figure 9).

While the determinants within NSP2 required for its RNA chaperone activity remain to be fully elucidated, Bravo et al. discovered that such activity also required the flexible CTR (residues 295–317) [64]. Using cryo-EM and structural proteomics studies, including hydrogen-deuterium exchange coupled with mass-spectrometry, the authors determined that the NSP2 CTR was not directly involved in RNA binding, a finding that had been previously reported [62,64]. Unexpectedly, the authors discovered that despite its reduced RNA chaperone activity, a mutant lacking the CTR (NSP2-ΔC) had enhanced capacity to unwind RNA stem-loops, and it was thus more efficient at destabilizing the RNA structure [64]. This result suggests that RNA helix unwinding by NSP2 must be fine-tuned in order for the protein to function as an efficient RNA chaperone. Indeed, the CTR harbors highly conserved negatively charged residues, which accelerate RNA dissociation from NSP2 [64]. Interestingly, these negatively charged residues appear to cluster next to the S313 residue involved in viroplasm formation [79]. In this manner, the CTR would acquire an extra negative charge due to phosphorylation, thus further promoting RNA dissociation via the charge repulsion mechanism. Taken together, the recent biophysical and structural data suggest that NSP2 may be a key RNA chaperone that relaxes intra-molecular RNA structure, increasing its propensity for inter-segment RNA base-pairing (Figure 9). This model is also in agreement with the results of in vitro RNA structure probing experiments that have recently confirmed the increased RNA backbone flexibility in the presence of NSP2 [97].

## 7. Future Directions

Taken together, the existing structural and functional studies of RV NSP2 have provided detailed mechanistic insights into the multifaceted roles that this protein plays during the viral replication cycle, particularly during early particle assembly. Notably, the current body of work strongly supports a critical role for octameric NSP2 in the biogenesis of viroplasms, which are cytoplasmic biomolecular condensates that serve as the sites of early particle assembly. However, there are several gaps in knowledge regarding how NSP2 nucleates viroplasms alongside NSP5 and other factors. For example, the conformational transition between the predominantly cytoplasmic dNSP2 and the viroplasm-localized vNSP2 remains unknown. Future studies could employ EM affinity grids and specific mAbs to capture dNSP2 vs. vNSP2 prior to cryo-EM single-particle reconstructions, thus revealing their different conformations [98]. Moreover, the role that NSP2 inter-octamer interactions play during viroplasm formation, if any, is not understood. Moving forward, it will be important to test whether NSP2 can mediate inter-octamer interactions in biologically relevant conditions. Approaches such as density gradient centrifugation, native gel electrophoresis and size-exclusion chromatography with multi-angle light scattering (SEC-MALS) could be used to detect the presence of NSP2 inter-octamer chains from lysates of infected cells and/or with recombinant protein. Mutagenesis could also be used to ablate the capacity of NSP2 to form inter-octamer chains in a manner that maintains other NSP2 interactions (e.g., with NSP5). Such experiments would be needed to unveil any effects of higher-ordered NSP2 multimerization on viroplasm biogenesis. Finally, studies seeking to identify other components that contribute to viroplasm formation (e.g., RNAs, cellular components and other viral proteins like VP2) will be important to pursue in future work. Quantitative in vitro reconstitution assays with individual interacting partners like that shown by Geiger et al. will likely prove valuable, as they would allow for direct probing of the sequence of events leading to the formation of these condensates [71].

In addition to its well-established role in viroplasm formation, the current literature also supports the notion that NSP2 acts as a viral RNA chaperone whereby it (i) binds viral +ssRNA, (ii) relaxes secondary structures via its helix-destabilizing activity and (iii) promotes +ssRNA assortment that may underpin the selective incorporation of the 11 RV genome segments. Still, the assortment/packaging signals within the +ssRNAs that allow for their selective enrichment into viroplasms and incorporation into particles are not known, and it remains a mystery how NSP2 binding to +ssRNA would reveal such signals [98]. Secondary structures within the +ssRNAs are beginning to be identified using biochemical approaches like SHAPE-Map [97]. Such experiments could be combined with the in vitro RNA–RNA interaction assay described by Borodavka et al. in order to validate which structures underpin selective assortment [96]. Of course, the functional significance of +ssRNA elements would need to be tested in the context of infected cells, perhaps by using small complimentary oligos that block specific RNA–RNA interactions.

In contrast to the well-established roles of NSP2 in viroplasm formation and +ssRNA assortment, the functional significance of NSP2 as an enzyme remains unclear. The NTPase/RTPase/NDP kinase enzymatic activities are dispensable for viroplasm formation as well as RNA chaperone activity, but they are clearly critical for viral replication. Understanding how these activities support RV replication has been difficult to tackle experimentally, but it is interesting to speculate about several possibilities. One simple explanation that has been put forth is that the NTPase/NDP kinase activities of NSP2 are critical for maintaining pools of nucleotides for use during viral RNA synthesis, which occurs inside viroplasms and in connection with early particle assembly. Specifically, −ssRNA strand synthesis (i.e., genome replication) occurs in the context of a core RI assembly intermediate containing NSP2. In this case, it is possible that the NTPase/NDP kinase activities of NSP2 ensure that the polymerase is being “fed” sufficient NTPs for −ssRNA strand synthesis. The observation that NSP2 has an RTPase activity might be an in vitro artifact of its NTPase functionality. However, it is alternatively possible that NSP2 plays a “moonlighting” role in helping RVs evade the host immune response. More specifically, RV −ssRNAs are reported to lack a 5′ γ-phosphate, which is a potent activator of RIG-I and, thus, the interferon pathway of the host cell [99,100]. Recombinant NSP2 was reported to bind VP1 near the dsRNA exit tunnel, at least in vitro, which could theoretically bring the RTPase in proximity with the 5′ end of −ssRNA for γ-phosphate removal [101]. Nevertheless, enzymatic NSP2 could have a more direct function in the regulation of −ssRNA synthesis. NSP2 has been reported to have an inhibitory effect on VP1/VP2-mediated dsRNA synthesis in vitro, and it would be of interest to test whether the catalytic activity of NSP2 is required for this inhibition [102]. Future studies of NSP2 activities will also benefit from the fully plasmid reverse genetics system, but perhaps in combination with *trans*-complementation approaches, as NSP2 mutant viruses would likely have severe replication defects [103]. Altogether, such future work will enrich the already deep knowledge base of this critical and multifaceted NSP2 protein during the RV replication cycle. Such work may also serve to broadly inform an understanding of viral factory formation, viral enzymology and viral RNA chaperone activities, as well as provide a foundation for antiviral drug design.

## Data Availability

Not applicable.

## References

[1] Matthijnssens J., Attoui H., Bányai K., Brussaard C.P., Danthi P., Del Vas M., Dermody T.S., Duncan R., Fang Q., Johne R. (2022). ICTV Virus Taxonomy Profile: Sedoreoviridae 2022. J. Gen. Virol..

[2] Matthijnssens J., Otto P.H., Ciarlet M., Desselberger U., Van Ranst M., Johne R. (2012). VP6-sequence-based cutoff values as a criterion for rotavirus species demarcation. Arch. Virol..

[3] Mihalov-Kovács E., Gellért Á., Marton S., Farkas S.L., Fehér E., Oldal M., Jakab F., Martella V., Bányai K. (2015). Candidate new rotavirus species in sheltered dogs, hungary. Emerg. Infect. Dis..

[4] Troeger C., Khalil I.A., Rao P.C., Cao S., Blacker B.F., Ahmed T., Armah G., Bines J.E., Brewer T.G., Colombara D.V. (2018). Rotavirus Vaccination and the Global Burden of Rotavirus Diarrhea Among Children Younger Than 5 Years. JAMA Pediatr..

[5] Díaz Alarcón R.G., Liotta D.J., Miño S. (2022). Zoonotic RVA: State of the Art and Distribution in the Animal World. Viruses.

[6] Arnold M., Patton J.T., McDonald S.M. (2009). Culturing, storage, and quantification of rotaviruses. Curr. Protoc. Microbiol..

[7] Settembre E.C., Chen J.Z., Dormitzer P.R., Grigorieff N., Harrison S.C. (2011). Atomic model of an infectious rotavirus particle. EMBO J..

[8] Jenni S., Salgado E.N., Herrmann T., Li Z., Grant T., Grigorieff N., Trapani S., Estrozi L.F., Harrison S.C. (2019). In situ Structure of Rotavirus VP1 RNA-Dependent RNA Polymerase. J. Mol. Biol..

[9] Dormitzer P.R., Greenberg H.B., Harrison S.C. (2001). Proteolysis of monomeric recombinant rotavirus VP4 yields an oligomeric VP5* core. J. Virol..

[10] Kumar D., Yu X., Crawford S.E., Moreno R., Jakana J., Sankaran B., Anish R., Kaundal S., Hu L., Estes M.K. (2020). 2.7 A cryo-EM structure of rotavirus core protein VP3, a unique capping machine with a helicase activity. Sci. Adv..

[11] Ding K., Celma C.C., Zhang X., Chang T., Shen W., Atanasov I., Roy P., Zhou Z.H. (2019). In situ structures of rotavirus polymerase in action and mechanism of mRNA transcription and release. Nat. Commun..

[12] Estes M., Kapikian A.Z., Knipe D.M., Howley P.M. (2007). Rotaviruses and Their Replication. Fields Virology.

[13] Davidson G.P., Goller I., Bishop R.F., Townley R.R., Holmes I.H., Ruck B.J. (1975). Immunofluorescence in duodenal mucosa of children with acute enteritis due to a new virus. J. Clin. Pathol..

[14] Starkey W.G., Collins J., Wallis T.S., Clarke G.J., Spencer A.J., Haddon S.J., Osborne M.P., Candy D.C.A., Stephen J. (1986). Kinetics, tissue specificity and pathological changes in murine rotavirus infection of mice. J. Gen. Virol..

[15] Arias C.F., López S. (2021). Rotavirus cell entry: Not so simple after all. Curr. Opin. Virol..

[16] Díaz-Salinas M.A., Romero P., Espinosa R., Hoshino Y., López S., Arias C.F. (2013). The Spike Protein VP4 Defines the Endocytic Pathway Used by Rotavirus To Enter MA104 Cells. J. Virol..

[17] Gutiérrez M., Isa P., Martin C.S.-S., Pérez-Vargas J., Espinosa R., Arias C.F., López S. (2010). Different Rotavirus Strains Enter MA104 Cells through Different Endocytic Pathways: The Role of Clathrin-Mediated Endocytosis. J. Virol..

[18] Trask S.D., McDonald S.M., Patton J.T. (2012). Structural insights into the coupling of virion assembly and rotavirus replication. Nat. Rev. Microbiol..

[19] Salgado E.N., Rodriguez B.G., Narayanaswamy N., Krishnan Y., Harrison S.C. (2018). Visualization of Calcium Ion Loss from Rotavirus during Cell Entry. J. Virol..

[20] Ludert J., Michelangeli F., Gil F., Liprandi F., Esparza J. (1987). Penetration and uncoating of rotaviruses in cultured cells. Intervirology.

[21] Lawton J.A., Estes M.K., Prasad B.V. (2000). Mechanism of genome transcription in segmented dsRNA viruses. Adv. Virus Res..

[22] Patton J.T., Chen D. (1999). RNA-binding and capping activities of proteins in rotavirus open cores. J. Virol..

[23] Liu M., Mattion N.M., Estes M.K. (1992). Rotavirus VP3 expressed in insect cells possesses guanylyltrans-ferase activity. Virology.

[24] Pizarro J.L., Sandino A.M., Pizarro J.M., Fernandez J., Spencer E. (1991). Characterization of rotavirus guan-ylyltransferase activity associated with polypeptide VP3. J. Gen. Virol..

[25] Vende P., Piron M., Castagne N., Poncet D. (2000). Efficient translation of rotavirus mRNA requires simul-taneous interaction of NSP3 with the eukaryotic translation initiation factor eIF4G and the mRNA 3′ end. J. Virol..

[26] López S., Oceguera A., Sandoval-Jaime C. (2016). Stress Response and Translation Control in Rotavirus Infection. Viruses.

[27] Mitzel D.N., Weisend C.M., White M.W., Hardy M.E. (2003). Translational regulation of rotavirus gene ex-pression. J. Gen. Virol..

[28] Papa G., Borodavka A., Desselberger U. (2021). Viroplasms: Assembly and Functions of Rotavirus Replication Factories. Viruses.

[29] Patton J.T., Silvestri L.S., Tortorici M.A., Vasquez-Del Carpio R., Taraporewala Z.F. (2006). Rotavirus genome replication and morphogenesis: Role of the viroplasm. Curr. Top. Microbiol. Immunol..

[30] Taraporewala Z.F., Patton J.T. (2004). Nonstructural proteins involved in genome packaging and replication of rotaviruses and other members of the Reoviridae. Virus Res..

[31] McNulty M.S., Curran W.L., McFerran J.B. (1976). The morphogenesis of a cytopathic bovine rotavirus in madin-darby bovine kidney cells. J. Gen. Virol..

[32] Gardet A., Breton M., Fontanges P., Trugnan G., Chwetzoff S. (2006). Rotavirus spike protein vp4 binds to and remodels actin bundles of the epithelial brush border into actin bodies. J. Virol..

[33] Ericson B.L., Graham D.Y., Mason B.B., Estes M.K. (1982). Identification, synthesis, and modifications of simian rotavirus SA11 polypeptides in infected cells. J. Virol..

[34] Mason B.B., Graham D.Y., Estes M.K. (1983). Biochemical mapping of the simian rotavirus SA11 genome. J. Virol..

[35] Both G.W., Bellamy A.R., Street J.E., Siegman L.J. (1982). A general strategy for cloning double-stranded RNA: Nucleotide sequence of the Simian-11 rotavirus gene 8. Nucleic Acids Res..

[36] Petrie B.L., Greenberg H.B., Graham D.Y., Estes M.K. (1984). Ultrastructural localization of rotavirus antigens using colloidal gold. Virus Res..

[37] Patton J.T., Gallegos C.O. (1988). Structure and protein composition of the rotavirus replicase particle. Virology.

[38] Gallegos C.O., Patton J.T. (1989). Characterization of rotavirus replication intermediates: A model for the assembly of single-shelled particles. Virology.

[39] Aponte C., Poncet D., Cohen J. (1996). Recovery and characterization of a replicase complex in rota-virus-infected cells by using a monoclonal antibody against NSP2. J. Virol..

[40] Helmberger-Jones M., Patton J.T. (1986). Characterization of subviral particles in cells infected with simian rotavirus SA11. Virology.

[41] McDonald S.M., Patton J.T. (2011). Assortment and packaging of the segmented rotavirus genome. Trends Microbiol..

[42] Mansell E.A., Patton J.T. (1990). Rotavirus RNA replication: VP2, but not VP6, is necessary for viral replicase activity. J. Virol..

[43] Patton J.T. (1996). Rotavirus VP1 alone specifically binds to the 3′ end of viral mRNA, but the interaction is not sufficient to initiate minus-strand synthesis. J. Virol..

[44] Patton J.T., Jones M.T., Kalbach A.N., He Y.W., Xiaobo J. (1997). Rotavirus RNA polymerase requires the core shell protein to synthesize the double-stranded RNA genome. J. Virol..

[45] McDonald S.M., Patton J.T. (2011). Rotavirus VP2 core shell regions critical for viral polymerase activation. J. Virol..

[46] Long C.P., McDonald S.M. (2017). Rotavirus genome replication: Some assembly required. PLoS Pathog..

[47] Gombold J.L., Estes M.K., Ramig R.F. (1985). Assignment of simian rotavirus SA11 temperature-sensitive mutant groups B and E to genome segments. Virology.

[48] Ramig R.F. (1983). Isolation and genetic characterization of temperature-sensitive mutants that define five additional recombination groups in simian rotavirus SA11. Virology.

[49] Ramig R.F., Petrie B.L. (1984). Characterization of temperature-sensitive mutants of simian rotavirus SA11: Protein synthesis and morphogenesis. J. Virol..

[50] Chen D., Gombold J.L., Ramig R.F. (1990). Intracellular RNA synthesis directed by temperature-sensitive mutants of simian rotavirus SA11. Virology.

[51] Kattoura M.D., Clapp L.L., Patton J.T. (1992). The rotavirus nonstructural protein, NS35, possesses RNA-binding activity in vitro and in vivo. Virology.

[52] Kattoura M.D., Chen X., Patton J.T. (1994). The rotavirus RNA-binding protein NS35 (NSP2) forms 10S multimers and interacts with the viral rna polymerase. Virology.

[53] Taraporewala Z., Chen D., Patton J.T. (1999). Multimers formed by the rotavirus nonstructural protein NSP2 bind to RNA and have nucleoside triphosphatase activity. J. Virol..

[54] Taraporewala Z.F., Schuck P., Ramig R.F., Silvestri L., Patton J.T. (2002). Analysis of a Temperature-sensitive mutant rotavirus indicates that NSP2 octamers are the functional form of the protein. J. Virol..

[55] Schuck P., Taraporewala Z., McPhie P., Patton J.T. (2001). Rotavirus nonstructural protein NSP2 self-assembles into octamers that undergo ligand-induced conformational changes. J. Biol. Chem..

[56] Taraporewala Z.F., Patton J.T. (2001). Identification and characterization of the Helix-destabilizing activity of rotavirus nonstructural protein NSP2. J. Virol..

[57] Afrikanova I., Fabbretti E., Burrone O.R., Miozzo M.C. (1998). Rotavirus NSP5 phosphorylation is up-regulated by interaction with NSP2. J. Gen. Virol..

[58] Fabbretti E., Afrikanova I., Vascotto F., Burrone O.R. (1999). Two non-structural rotavirus proteins, NSP2 and NSP5, form viroplasm-like structures in vivo. J. Gen. Virol..

[59] Jayaram H., Taraporewala Z., Patton J.T., Prasad B.V.V. (2002). Rotavirus protein involved in genome replication and packaging exhibits a HIT-like fold. Nature.

[60] Jiang X., Jayaram H., Kumar M., Ludtke S.J., Estes M.K., Prasad B.V.V. (2006). Cryoelectron microscopy structures of rotavirus NSP2-NSP5 and NSP2-RNA complexes: Implications for genome replication. J. Virol..

[61] Kumar M., Jayaram H., Vasquez-Del Carpio R., Jiang X., Taraporewala Z.F., Jacobson R.H., Patton J.T., Prasad B.V. (2007). Crystallographic and biochemical analysis of rotavirus NSP2 with nucleotides reveals a nucleoside diphosphate kinase-like activity. J. Virol..

[62] Hu L., Chow D.-C., Patton J.T., Palzkill T., Estes M.K., Prasad B.V.V. (2012). Crystallographic Analysis of Rotavirus NSP2-RNA Complex Reveals Specific Recognition of 5′ GG Sequence for RTPase Activity. J. Virol..

[63] Criglar J.M., Anish R., Hu L., Crawford S.E., Sankaran B., Prasad B.V.V., Estes M.K. (2018). Phosphorylation cascade regulates the formation and maturation of rotaviral replication factories. Proc. Natl. Acad. Sci. USA.

[64] Bravo J.P.K., Bartnik K., Venditti L., Acker J., Gail E.H., Colyer A., Davidovich C., Lamb D.C., Tuma R., Calabrese A.N. (2021). Structural basis of rotavirus RNA chaperone displacement and RNA annealing. Proc. Natl. Acad. Sci. USA.

[65] Carpio R.V.-D., Gonzalez-Nilo F.D., Riadi G., Taraporewala Z.F., Patton J.T. (2006). Histidine triad-like motif of the rotavirus NSP2 Octamer mediates both RTPase and NTPase activities. J. Mol. Biol..

[66] Martin D., Duarte M., Lepault J., Poncet D. (2010). Sequestration of free tubulin molecules by the viral protein NSP2 induces microtubule depolymerization during rotavirus infection. J. Virol..

[67] Eichwald C., Rodriguez J.F., Burrone O.R. (2004). Characterization of rotavirus NSP2/NSP5 interactions and the dynamics of viroplasm formation. J. Gen. Virol..

[68] Carreño-Torres J.J., Gutiérrez M., Arias C.F., López S., Isa P. (2010). Characterization of viroplasm formation during the early stages of rotavirus infection. Virol. J..

[69] Eichwald C., Arnoldi F., Laimbacher A.S., Schraner E.M., Fraefel C., Wild P., Burrone O.R., Ackermann M. (2012). Rotavirus viroplasm fusion and perinuclear localization are dynamic processes requiring stabilized microtubules. PLoS ONE.

[70] Nichols S.L., Nilsson E.M., Brown-Harding H., LaConte L.E.W., Acker J., Borodavka A., Esstman S.M. (2023). Flexibility of the Rotavirus NSP2 C-Terminal Region Supports Factory Formation via Liquid-Liquid Phase Separation. J. Virol..

[71] Geiger F., Acker J., Papa G., Wang X., Arter W.E., Saar K.L., Erkamp N.A., Qi R., Bravo J.P., Strauss S. (2021). Liquid-liquid phase separation underpins the formation of replication factories in rotaviruses. EMBO J..

[72] Strauss S., Acker J., Papa G., Desiro D., Schueder F., Borodavka A., Jungmann R. (2023). Principles of RNA recruitment to viral ribonucleoprotein condensates in a segmented dsRNA virus. eLife.

[73] Dhillon P., Tandra V.N., Chorghade S.G., Namsa N.D., Sahoo L., Rao C.D. (2018). Cytoplasmic Relocalization and Colocalization with Viroplasms of Host Cell Proteins, and Their Role in Rotavirus Infection. J. Virol..

[74] López T., Rojas M., Ayala-Bretón C., López S., Arias C.F. (2005). Reduced expression of the rotavirus NSP5 gene has a pleiotropic effect on virus replication. J. Gen. Virol..

[75] Silvestri L.S., Taraporewala Z.F., Patton J.T. (2004). Rotavirus replication: Plus-sense templates for double-stranded RNA synthesis are made in viroplasms. J. Virol..

[76] Carpio R.V.-D., González-Nilo F.D., Jayaram H., Spencer E., Prasad B.V.V., Patton J.T., Taraporewala Z.F. (2004). Role of the histidine triad-like motif in nucleotide hydrolysis by the rotavirus RNA-packaging protein NSP2. J. Biol. Chem..

[77] Taraporewala Z.F., Jiang X., Vasquez-Del Carpio R., Jayaram H., Prasad B.V., Patton J.T. (2006). Structure-function analysis of rotavirus NSP2 octamer by using a novel complementation system. J. Virol..

[78] Criglar J.M., Hu L., Crawford S.E., Hyser J.M., Broughman J.R., Prasad B.V.V., Estes M.K. (2014). A novel form of rotavirus NSP2 and phosphorylation-dependent NSP2-NSP5 interactions are associated with viroplasm assembly. J. Virol..

[79] Criglar J.M., Crawford S.E., Zhao B., Smith H.G., Stossi F., Estes M.K. (2020). A Genetically Engineered Rotavirus NSP2 Phosphorylation Mutant Impaired in Viroplasm Formation and Replication Shows an Early Interaction between vNSP2 and Cellular Lipid Droplets. J. Virol..

[80] Eichwald C., Jacob G., Muszynski B., Allende J.E., Burrone O.R. (2004). Uncoupling substrate and activation functions of rotavirus NSP5: Phosphorylation of Ser-67 by casein kinase 1 is essential for hyperphos-phorylation. Proc. Natl. Acad. Sci. USA.

[81] Criglar J.M., Estes M.K., Crawford S.E. (2022). Rotavirus-Induced Lipid Droplet Biogenesis Is Critical for Virus Replication. Front. Physiol..

[82] Crawford S.E., Desselberger U. (2016). Lipid droplets form complexes with viroplasms and are crucial for rotavirus replication. Curr. Opin. Virol..

[83] Cheung W., Gill M., Esposito A., Kaminski C.F., Courousse N., Chwetzoff S., Trugnan G., Keshavan N., Lever A., Desselberger U. (2010). Rotaviruses associate with cellular lipid droplet components to replicate in viroplasms, and compounds disrupting or blocking lipid droplets inhibit viroplasm formation and viral replication. J. Virol..

[84] Campagna M., Marcos-Villar L., Arnoldi F., de la Cruz-Herrera C.F., Gallego P., González-Santamaría J., González D., Lopitz-Otsoa F., Rodriguez M.S., Burrone O.R. (2013). Rotavirus viroplasm proteins interact with the cellular sumoylation system: Implications for viroplasm-like structure formation. J. Virol..

[85] Contin R., Arnoldi F., Campagna M., Burrone O.R. (2010). Rotavirus NSP5 orchestrates recruitment of viroplasmic proteins. J. Gen. Virol..

[86] Mohan K.V.K., Muller J., Atreya C.D., Campbell T.B., Schneider K., Wrin T., Petropoulos C.J., Connick E. (2003). The N- and C-terminal regions of rotavirus NSP5 are the critical determinants for the formation of viroplasm-like structures independent of NSP2. J. Virol..

[87] Buttafuoco A., Michaelsen K., Tobler K., Ackermann M., Fraefel C., Eichwald C. (2020). Conserved Rotavirus NSP5 and VP2 Domains Interact and Affect Viroplasm. J. Virol..

[88] Martin D., Ouldali M., Ménétrey J., Poncet D. (2011). Structural organisation of the rotavirus nonstructural protein NSP5. J. Mol. Biol..

[89] Afrikanova I., Miozzo M.C., Giambiagi S., Burrone O. (1996). Phosphorylation generates different forms of rotavirus NSP5. J. Gen. Virol..

[90] Eichwald C., Vascotto F., Fabbretti E., Burrone O.R. (2002). Rotavirus NSP5: Mapping phosphorylation sites and kinase activation and viroplasm localization domains. J. Virol..

[91] Poncet D., Lindenbaum P., L’Haridon R., Cohen J. (1997). In vivo and in vitro phosphorylation of rotavirus NSP5 correlates with its localization in viroplasms. J. Virol..

[92] Papa G., Venditti L., Arnoldi F., Schraner E.M., Potgieter C., Borodavka A., Eichwald C., Burrone O.R. (2019). Recombinant Rotaviruses Rescued by Reverse Genetics Reveal the Role of NSP5 Hyperphosphorylation in the Assembly of Viral Factories. J. Virol..

[93] Campagna M., Budini M., Arnoldi F., Desselberger U., Allende J.E., Burrone O.R. (2007). Impaired hyper-phosphorylation of rotavirus NSP5 in cells depleted of casein kinase 1alpha is associated with the for-mation of viroplasms with altered morphology and a moderate decrease in virus replication. J. Gen. Virol..

[94] Cabral-Romero C., Padilla-Noriega L. (2006). Association of rotavirus viroplasms with microtubules through NSP2 and NSP5. Memórias Inst. Oswaldo Cruz.

[95] Jing Z., Shi H., Chen J., Shi D., Liu J., Guo L., Tian J., Wu Y., Dong H., Zhang J. (2021). Rotavirus Viroplasm Biogenesis Involves Microtubule-Based Dynein Transport Mediated by an Interaction between NSP2 and Dynein Intermediate Chain. J. Virol..

[96] Borodavka A., Dykeman E.C., Schrimpf W., Lamb D.C. (2017). Protein-mediated RNA folding governs sequence-specific interactions between rotavirus genome segments. eLife.

[97] Coria A., Wienecke A., Knight M.L., Desiro D., Laederach A., Borodavka A. (2022). Rotavirus RNA chap-erone mediates global transcriptome-wide increase in RNA backbone flexibility. Nucleic Acids Res..

[98] Boudreaux C.E., Kelly D.F., McDonald S.M. (2015). Electron microscopic analysis of rotavirus assembly-replication intermediates. Virology.

[99] Imai M., Akatani K., Ikegami N., Furuichi Y. (1983). Capped and conserved terminal structures in human rotavirus genome double-stranded RNA segments. J. Virol..

[100] McCrae M., McCorquodale J. (1983). Molecular biology of rotaviruses *V. terminal* structure of viral RNA species. Virology.

[101] Viskovska M., Anish R., Hu L., Chow D.-C., Hurwitz A.M., Brown N.G., Palzkill T., Estes M.K., Prasad B.V.V. (2014). Probing the sites of interactions of rotaviral proteins involved in replication. J. Virol..

[102] Vende P., Tortorici M., Taraporewala Z.F., Patton J.T. (2003). Rotavirus NSP2 interferes with the core lattice protein VP2 in initiation of minus-strand synthesis. Virology.

[103] Kanai Y., Komoto S., Kawagishi T., Nouda R., Nagasawa N., Onishi M., Matsuura Y., Taniguchi K., Kobayashi T. (2017). Entirely plasmid-based reverse genetics system for rotaviruses. Proc. Natl. Acad. Sci. USA.

